# Construction and identification of the recombinant plasmid pET30a-EgA31-Eg95 of *Echinococcus granulosus*

**DOI:** 10.3892/etm.2013.1393

**Published:** 2013-11-07

**Authors:** YANHUA LI, FENGBO ZHANG, MOHAMMED H. ALTHUNAYAN, XIAO-AN HU, YAN XIN, HAIYING JIA, YUYUAN GUO, XIUMIN MA, HAO WEN, JIANBING DING

**Affiliations:** 1 Xinjiang Laboratory of Hydatid Fundamental Medicine, The First Affiliated Hospital, Xinjiang Medical University, Urumqi, Xinjiang 830054, P.R. China; 2 Department of Immunology, College of Basic Medicine of Xinjiang Medical University, Urumqi, Xinjiang 830054, P.R. China

**Keywords:** *Echinococcus granulosus*, EgA31-Eg95 antigen gene, prokaryotic expression plasmid

## Abstract

To clone the Eg95 and EgA31 antigen genes into the prokaryotic expression plasmid pET30a-EgA31-Eg95, we expressed the recombinant protein EgA31-Eg95 and confirmed with western blot analysis. The total RNA was extracted from the protoscoleces of *Echinococcus granulosus* (*E. granulosus*) adult worms. The complementary DNA (cDNA) encoding the EgA31 antigen was amplified via quantitative real-time polymerase chain reaction (qPCR). The recombinant plasmid pET30a-EgA31 was used as a carrier and was connected with the Eg95 vector. The recombinant plasmid pET30a-EgA31-Eg95 was constructed and the fusion protein EgA31-Eg95 was detected using sodium dodecyl sulfate-polyacrylamide gel electrophoresis (SDS-PAGE). The positive clone was the empty recombinant vector. The recombinant protein pET30a-EgA31-Eg95 was ~46 kDa, and the expressed product accounted for approximately 20% of the total soluble proteins. We successfully constructed the recombinant plasmid pET30a-EgA31-Eg95 and expressed the recombinant protein EgA31-Eg95. The results may be the foundation of research on its immunogenicity in the future.

## Introduction

*Echinococcus granulosus* (*E. granulosus*) causes cystic echinococcosis (CE), which seriously injures human health and delays the development of the animal breeding industry. China is one of the countries with a high rate of *E. granulosus* disease (1,2), which is mainly common in the west pastoral and semi-pastoral areas of China, including Xinjiang, Qinghai and Gansu (3). The main treatment of hydatid disease is surgery supplemented by drug treatment (4). However, the surgery is harmful to the human body, whereas a molecular vaccine is an ideal method to prevent alveolar echinococcosis (5,6). Since *E. granulosus* is a multicellular parasite and the antigen structure is highly complex, it is necessary to study the *E. granulosus* protoscolex antigen and the adult worm antigen to identify the protein for a polyvalent vaccine.

The EgA31 antigen was currently studied as the dominant antigen of the adult worm. If the rostellum of the protoscolex entering the host intestine is not removed or absorbed by the intestinal mucosa, the worms are excreted out of the host (7). Fraize *et al*(8) built a model *in vitro* to evaluate the T cell response of the final host to the protoscolex and Eg antigen. The study observed that the protoscolex did not cause changes in cytokine production, which was consistent with the Eg metacestode showing no or little immunogenicity within the final host (9). This was consistent with Vuitton’s (10) theory, i.e. that the parasites are able to shed the surface antigen and interfere with antigen presentation mechanisms to reduce immunogenicity and evade the immune system of the host. EgA31 antigen may increase interleukin-10 (IL-10) and IL-12 production. IL-12 may promote the production of interferon-γ (IFN-γ) and inhibit protective immunity, resulting in chronic infection (11).

Eg95 protein is an ideal protective antigen and is one of the most extensively studied antigenic components. The Eg95 recombinant protein vaccine immunized the intermediate host (sheep), and 86% complete immune protection was obtained (6). Furthermore, Ding *et al*(12) and Liu *et al*(13) demonstrated that the pcDNA3-Eg95 gene vaccine and the Eg95 recombinant protein were able to produce specific humeral and cellular immune responses in mice. Alvite and Esteves (14) observed that the expression of the EgA31 antigen may be correlated with the sucking function of the scolex in each growth stage of the adult. This antigen is located at multiple sites on the parasite protoscolex and adult worms. Saboulard *et al*(15) observed that EgA31 protein exhibited a high level of antigenicity and immunogenicity. EgA31 was also demonstrated to show the strongest immunoreactivity in a different study (16). It is possible to develop a protein composition vaccine; thus, the Eg95 and EgA31 antigens were selected as vaccine candidates and recombinant antigens, and were predicted to enhance the immune response and play a significant role in immunogenicity. The aim was to provide further experimental foundation for a multivalent EgA31-Eg95 vaccine.

## Materials and methods

### E. granulosus protoscolex and adult specimens, serum, plasmids and strains

The *E. granulosus* protoscoleces were obtained from the slaughterhouse (Urumqi, China) from *E. granulosus* infection to liver cysts, while the adult specimens were obtained from infected canine intestine provided by the Veterinary Research Institute of the Xinjiang Academy of Animal Science and (Urumqi, China). *E. granulosus*-infected dog serum and healthy serum were also provided by the Veterinary Research Institute of the Xinjiang Academy of Animal Science. The mice infected with *Echinococcus granulosus* were supplied by Animal Center of Xinjiang Medical University. The cloning plasmid pUCm-T was purchased from MBI, Inc. (Pomona, CA, USA). The prokaryotic expression plasmid pET30a and the recombinant plasmids pET28a-Eg95 and pET30a-EgA31 were obtained from Xinjiang Laboratory of Hydatid Fundamental Medicine, First Affiliated Hospital of Xinjiang Medical University (Urumqi, China), while the *Escherichia coli* (*E. coli*) DH5-α was obtained from Invitrogen Life Technologies (Carlsbad, CA, USA). The *E. coli* strain BL21 (DE3) (Panvera, Madison, WI, USA) was used to amplify the recombinant vector. All patients and healthy controls signed the informed consent, the experimental design was approved by the ethical committee (Approval Number: 20120220-126). All experiments using mice were performed in accordance with protocols approved by Xinjiang Medical University Animal Ethics Committee according to China Guidelines on Animals Care (No. A-20100920002).

### Main reagents and formula

TRIzol^®^, DL2000 DNA Marker, λ*Hin*dIII digest, the restriction enzymes *Bam*HI, *Sac*I and *Not*I, and T4 DNA ligase were purchased from Takara Bio, Inc. (Shiga, Japan). A cDNA synthesis kit (MBI, Inc.) was used to amplify the AMV reverse transcriptase, while Taq DNA polymerase and pUCm-T (MBI, Inc.) were used to amplify the genes. Prestained protein marker and a Bicinchoninic acid (BCA) Protein Quantitation kit from BioTeke Corp. (Beijing, China) were used in the western blotting. Goat anti-rabbit immunoglobulin G (IgG)-horseradish peroxidase (HRP) and goat anti-human IgG-HRP were obtained from Sigma (St. Louis, MO, USA). In addition, a UNIQ-10 Column Mini Plasmid kit and UNIQ-10 Column DNA Gel Extraction kit (Bio-Rad Laboratories (Shanghai) Co., Ltd., Shanghai, China) were used.

### Primer design and synthesis

According to the Echinococcus EgA31 gene sequence (GenBank Accession: AF067807), Eg95 gene sequence (GenBank Accession: X90928) and Prokaryotic expression plasmid pET30a restriction map, we designed two pairs of primers with the software. Two pairs of primers were designed with the DNAman software (Lynnon Corp., Pointe-Claire, QC, Canada), and synthesized by Sangon (Shanghai, China). The primers were as follows: EgA31 forward primer P1, 5′-**GGA TCC** CGT CTA AGA ATA TCT GCA GCT GA-3′ (bold sequence was the restriction site of *Bam*HI) and reverse primer P2, 5′-**GAG CTC** AGT CTC AGC CCT TGT TTC AAG CA-3′ (bold for the restriction site of *Sac*I); Eg95 upstream primer P3, 5′-**GAG CTC** ATG GCA TTC CAG TTA TGT CT-3′ (bold nucleotides represent the *Sac*I restriction site) and reverse primer P4, 5′-**GCG GCC GC**C AGT GCT TTC CTT CTT-3′ (bold nucleotides represent the *Not*I restriction site).

### Transcription and reverse extraction of the total RNA of E. granulosus protoscolex and adult specimens

The total RNA was extracted from the liver of mice using TRIzol reagent. A transcription kit was used to amplify all mRNA into complementary DNA (cDNA).

### Cloning and identification of the EgA31- and Eg95-encoding genes

The cDNA in adult specimens was used as a template to clone the EgA31 target fragment using reverse transcription-polymerase chain reaction (RT-PCR). The cDNA in the protoscolex specimens was used as a template for the Eg95-encoding gene to clone the Eg95 target fragment with an RT-PCR kit, according to the manufacturer’s instructions (Invitrogen Life Technologies).

### Construction of the prokaryotic expression plasmid pET30a-EgA31-Eg95

**Figure 1:**
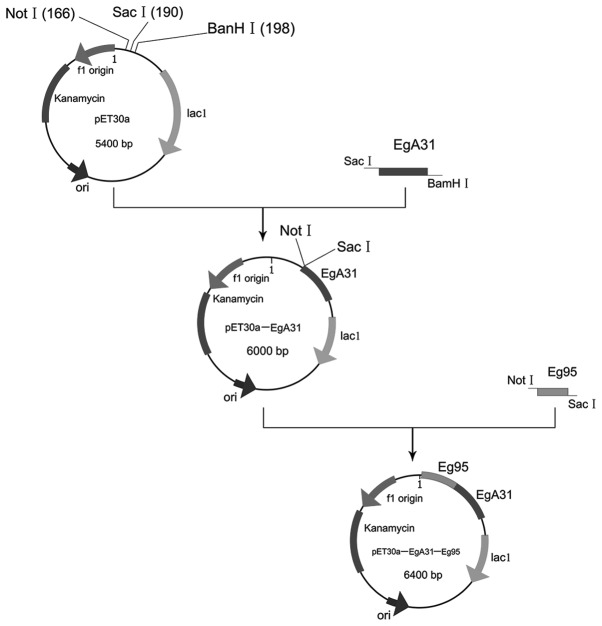
Diagram of the constructed recombinant plasmid pET30a-EgA31-Eg95.

Plasmid construction is shown schematically in Fig. 1. Genetic engineering and cloning were used to channel the Eg95 antigen gene into the shuttle plasmid pET30a-EgA31 to build the recombinant plasmid pET30a-EgA31-Eg95 with two sections of the target gene. The recombinant plasmids pET30a-EgA31 and pUCm-T/Eg95 were extracted. The double digestion of the recombinant plasmid pET30a-EgA31 consisted of a total reaction volume of 160 μl, which included the following: Plasmid 16 μl, 8 U/μl *Sac*I 5 μl, 10 U/μl *Not*I 4 μl, 10X K buffer 8 μl, 0.1% bovine serum albumin (BSA) 16 μl and ddH_2_O 111 μl. The double digestion of the recombinant plasmid pUCm-T/Eg95 consisted of a total reaction volume of 200 μl, which included the following: Plasmid 20 μl, 8 U/μl *Sac*I 6 μl, *Not*I 5 μl 10 U/μl, 10X K buffer 10 μl, 0.1% BSA 20 μl and ddH_2_O 139 μl. The digestions were performed at 37°C for 12 h. Following separation by 1.2% agarose gel, the corresponding fragment of target gene Eg95 and a large fragment of linear pET30a were recycled by a DNA gel extraction kit. The DNA was then dissolved in ddH_2_O.

The two digested plasmids were linked. Subsequently, the recombinant plasmid pET30a-EgA31-Eg95 was identified via sequencing: P1 and P4 were amplified (annealing temperature 55°C). The restriction enzyme digestion was used to confirm the amplified DNA and affirm that the sites were correct (*Bam*HI, *Not*I digestion). The recombinant plasmid pET30a-EgA31-Eg95 was sequenced to confirm its identity. The measurement analysis was performed with a kit purchased from Sangon.

### Expression and purification of the recombinant protein

#### Recombinant Eg95 protein

The prokaryotic expression plasmid pET28a-Eg95 was transformed into *E. coli* BL21 (DE3), and 2% of the inoculation amount of the overnight culture of a single bacterium was transferred to liquid LB medium containing kanamycin. The A600 absorbance value was ~0.6. Protein expression was induced with the final concentration of 0.1 mmol/l isopropylthio-β-galactoside (IPTG) at 28 and 37°C. The samples were collected at different induction times (0, 1, 2, 3, 4, 5 and 6 h) and bacteria were obtained. The samples were then placed into a boiling water bath for 5 min and 12% sodium dodecyl sulfate-polyacrylamide gel electrophoresis (SDS-PAGE) was performed to assess expression.

#### Recombinant EgA31 and EgA31-Eg95 protein

The recombinant plasmid pET30a-EgA31-Eg95 1 μl and *E. coli* BL21 (DE3) competent cells transformed by pET30a-EgA31-Eg95 1 μl were taken for transformation into *E. coli* BL21 (DE3)-competent cells, and PCR was used to screen the recombinants. Cultured and expression-induced thalli were collected at 0, 2, 4 and 6 h using SDS-PAGE (pET30a-EgA31-Eg95 recombinant protein was detected using SDS-PAGE Mini Protein IH using a miniature protein electrophoresis system). The recombinant protein was purified by a His column using chromatographic purification of the target protein. Protease inhibitor (Ben 15 μg/ml, Leu 2 μg/ml, PMSF 1 mmol/l, Pep 1 μg/ml) was added to 200 ml of bacterial culture following induction for 3 h. The cells were lysed by ice bath sonication and centrifugation, and the purified recombinant pET28a-Eg95 protein was obtained by His-Bind Resin, SDS-PAGE electrophoresis analysis.

#### Recombinant protein detection

The separation gel was retained for transformation to a membrane, with a constant current of 120 mA at 4°C overnight. The gel was sealed and agitated at 37°C for 2 h. The primary antibody was added and incubated at 37°C for 2 h. The nitrocellulose membrane was placed in the diluted secondary antibody, with stable shaking at 37°C for an hour. 3,3′-Diaminobenzidine (DAB) staining was performed prior to rinsing with water. Dog serum infected with *E. granulosus* (1:100 dilution) was used as the primary antibody to EgA31 recombinant protein and recombinant EgA31-Eg95 protein, and the secondary antibody was HRP-labeled rabbit anti-dog IgG (1:400 dilution with phosphate-buffered saline with Tween 20).

## Results

### Total RNA extraction from E. granulosus protoscolex and adult

**Figure 2:**
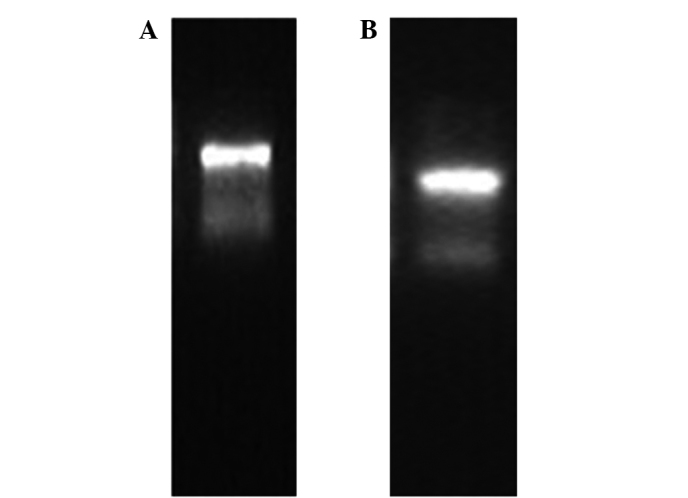
Agarose gel electrophoresis result of the *E. granulosus* protoscoleces and adult worms total RNA expression. The mRNA levels were high. (A) *E. granulosus* protoscoleces total RNA, (B) *E. granulosus* adult worms total RNA.

The total RNA was run in 1.2% 3-(N-morpholino)propanesulfonic acid (MOPS)-formaldehyde denaturing gel electrophoresis (Fig. 2). The density of the RNA bands was measured by the absorption at wavelengths of 260 and 280 nm with a nucleic acid and protein valuating machine (NanoDrop 2000; Thermo Scientific, Waltham, MA, USA). The A260/A280 ratio for protoscolex RNA was 1.81 and 1.85 for adults. The total RNA extraction was successful and the purity and concentration of RNA were high.

### Cloning of EgA31 and Eg95 antigen genes

**Figure 3:**
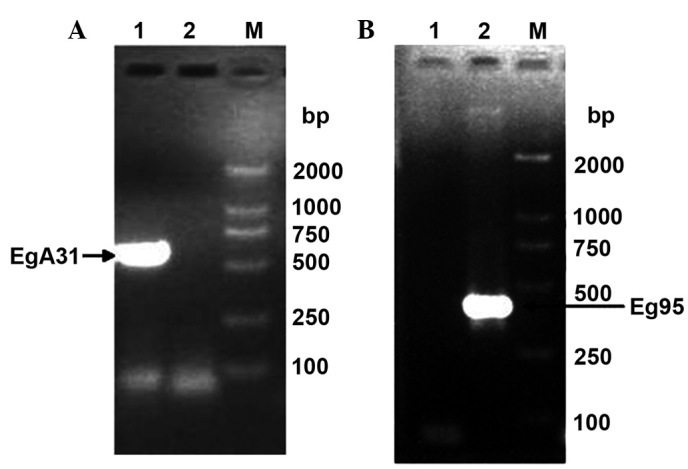
Quantitative real-time polymerase chain reaction (qPCR) products of EgA31and Eg95 gene. (A) Lane 1: qPCR products of EgA31, Lane 2: negative control without template. (B) Lane 1: negative control without template, Lane 2: qPCR products of Eg95. M, DL2000 DNA marker.

Using adult *E. granulosus* cDNA and *E. granulosus* protoscolex cDNA as templates, respectively, EgA31 primers and Eg95 primers were used for RT-PCR amplification. The PCR products were analyzed using 1.2% agarose gel electrophoresis, and specific bands showed at 636 and 402 bp (Fig. 3), which were consistent with the expected results. The negative control without template showed no specific band, demonstrating that the amplification of the EgA31 and Eg95 gene fragments was successful.

### EgA31 and Eg95 antigen gene sequences

A comparison between the recorded or registered EgA31 antigen gene sequence in GenBank (accession no. AF067807) and the cloning EgA31 antigen-specific sequence showed that the two sequences were identical. The cloned Eg95 antigen gene sequence was also consistent with the Eg95 antigen gene sequence in GenBank (accession no. X90928).

### Construction of the prokaryotic expression plasmid pET30a-EgA31-Eg95

#### Identification of enzyme digestion and amplification

**Figure 4:**
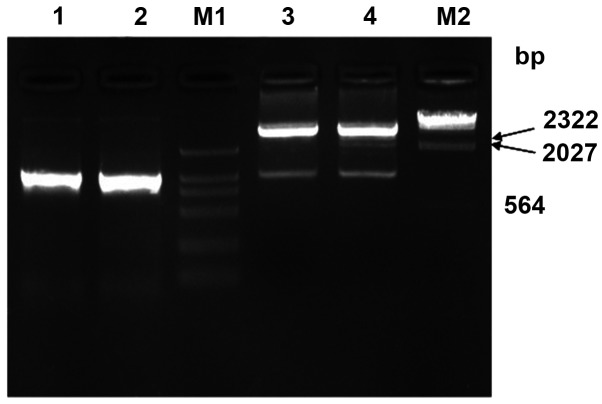
Identification of pET30a-EgA31-Eg95 recombinant plasmid by polymerase chain reaction (PCR) and digestion with restriction enzymes. M1: DL2000 DNA Marker; Lanes 1 and 2: PCR products of EgA31-Eg95; Lanes 3 and 4: recombinant plasmid digested by *Bam*HI/*Not*I; M2: λ*Hin*dIII.

Eg95 antigen-targeted gene fragments were obtained at 402 bp and a pET30a-EgA31 fragment at 5.5 kbp. Eg95 fragment and pET30a-EgA31 fragment were recycled by electrophoresis and connected by T4 DNA ligase, directionally cloned pET30a-EgA31-Eg95 vector through prokaryotic expression, following the process of transformation, culturing and extraction of small amount of plasmid. The 1038 bp EgA31-Eg95 DNA fragment was obtained by either PCR method or recombinant plasmid pET30a-EgA31-Eg95 digestion with *Bam*HI and *Not*I, consistent with expected products (Fig. 4).

#### Sequencing analysis

The digested identified recombinant plasmid broth (1 ml) was sent to Sangon for sequencing. Due to the connected fragment length of 1,038 bp, bidirectional sequencing was used. The cloned EgA31-Eg95 antigen gene was identical to the EgA31 and Eg95 cDNA sequences from the gene library, encoding 346 amino acids. The molecular weight of the recombinant protein was 31 kDa.

#### Western blotting results of EgA31-Eg95 recombinant protein

**Figure 5:**
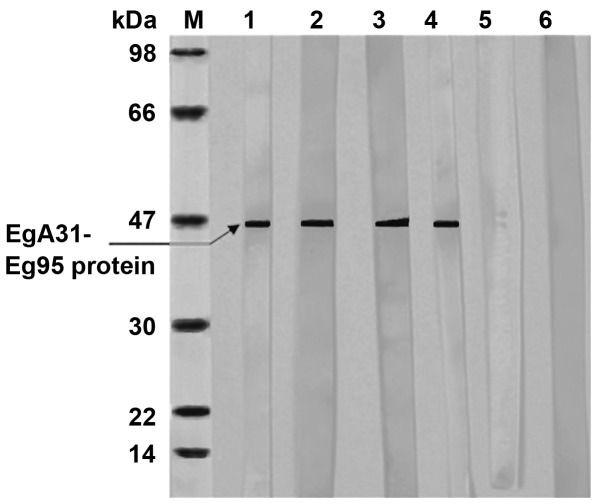
Western blot analysis results of the recombinant EgA31-Eg95. M, DL2000 DNA marker. Lanes 1–4: dog sera samples infected with Eg, showed a positive reaction to EgA31-Eg95 antigen; Lanes 5 and 6: two normal sera samples (negative control) did not show any significant reaction.

The correctly identified recombinant expression vector pET30a-EgA31-Eg95 was detected using 12% SDS-PAGE, which showed that its size was consistent with the expected protein size. The induced recombinant protein EgA31-Eg95 was transferred to a nitrocellulose membrane to combine with the corresponding antibody (the primary antibody) in the serum of dogs infected with *E. granulosus*, to form antigen-antibody complexes. The primary antibody then combined with the HRP-labeled antibody (the secondary antibody), resulting in the complex also being labeled with HRP. Following a chromogenic reaction, the recombinant antigen proteins EgA31-Eg95 were colored. The results are shown in Fig. 5.

## Discussion

The immune response caused by hydatid infection is complex and diverse, including humeral and cellular immunity, and the involvement of other cells and the complement system. Different mechanisms of protective immunity are induced by different antigens, and the protective antigen specificity is also different at various stages. During the long-term co-evolution of hydatid and host, a variety of immune evasion mechanisms were induced (17). This is the reason that the ideal effect of the hydatid monovalent vaccine is difficult to achieve. A combined immunization program was used, aiming at different developmental stages or different parts of *Schistosoma japonicum* and selecting antigens from different sources, with the aim of producing a synergistic effect by different immune mechanisms (18). This was performed in order to overcome the problems of the low-level immune protection induced by single antigen molecules and to enhance the immune effect of the vaccine. This is likely to represent a novel direction in the development of an anti-hydatid vaccine (19).

The amino acid sequence analysis of EgA31 antigen gene showed a 20–30% homology with flat phylum worm paramyosin and myosin, containing epitopes recognized by specific IgE (20,21). In this study, according to the screening for the kanamycin resistance marker gene of the pET30a vector, positive clones were sent for sequencing following PCR amplification and correct restriction enzyme digestion. The sequencing results showed that the selected positive clones were positive connection recombinants. The cloned EgA31 antigen gene was identical to the EgA31 cDNA sequence in the gene library, located between 493 and 1386 bp of the full-length sequences and encoding 212 amino acids. Sequence analysis showed that pET30a, which expresses in the form of the 6X His-EgA31 fusion protein, contained 280 amino acids, and that the molecular weight of the recombinant protein should be 31 kDa. This was consistent with the SDS-PAGE results. The data showed that the mixed EgA31 antigens [EgA31, EgTrp and fatty acid-binding protein 1 (FABP1)] caused higher levels of cytokines than the use of mono-EgA31 antigen. EgA31 antigen belongs to a protein family that confers protective immunity against numerous worm infections (22).

The nucleotide sequencing showed that the cloned 5′ end in Eg95 was nine nucleotides longer than in Eg48. The Eg48 recombinant protein was used to immune sheep, the larva may be reduced of 83%, average amount of cysts was 26.6 (23), which suggests that there are more likely protective immunodominant epitopes within the nine nucleotides. However, the response between Eg95 recombinant protein and the serum of patients with CE has been rarely reported in China. The positive response of Eg95 protein and patient sera has an important role in this vaccine development. If the positive rate of Eg95 in the patient serum is high, then it may be considered as a postoperative treatment in addition to clinical services. Therefore, this study selected to combine the protective antigens Eg95 and EgA31 against Echinococcus infection.

The western blotting results showed that there were positive reactions to the EgA31 and EgA31-Eg95 antigens in the sera of infected dogs, whereas this reaction was not observed in normal serum. This showed that the obtained EgA31 and the EgA31-Eg95 fusion protein had good antigenicity and were antigen molecules with immunological activity. Induced EgA31 and EgA31-Eg95 protein may be used as heterologous antigen to immune animals (including rat and rabbit), to test their immune characteristics and evaluate whether EgA31-Eg95 protein has a high level of immunological protection as a candidate vaccine against Echinococcosis. Polyclonal antiserum or anti-EgA31 and anti-EgA31-Eg95 monoclonal antibodies (McAbs) were obtained by a hybridoma technique. These were able to be identified using western blotting to analyze the induced expression of the corresponding proteins produced by the transformed bacteria or cells. The anti-EgA31-Eg95 antibody in Eg-infected dog serum in the western blot analysis was able to be used as the positive control serum. The most basic role of the EgA31-Eg95 protein is as an antigen.

The results of this study showed that three recombinant plasmids pET30a-EgA31, pET30a-EgA31-Eg95 and pET28a-Eg95 were stably expressed in *E. coli*. Among the three factors of induction temperature, induction time and concentration, the effect of temperature and time on the expression of the fusion protein was not notable. The expression of pET30a-EgA31-Eg95 increased with the extension of induction time. We selected Eg95 antigen, which is expressed in the protoscolex and may protect the host against *Schistosoma mansoni* infection, and EgA31 antigen, which is expressed in the scolex, skin and subcutaneous muscle layer, as candidate vaccine molecules. Eg95 and EgA31 antigen genes were constructed into a multivalent vaccine, to stimulate the immune response of the host immune system against *E. granulosus* protoscolex and the adult worm, enabling the host to obtain effective immunological protection. This may provide a wide application prospect for the design and development of an Echinococcosis vaccine.

## Abbreviations

*E. granulosus*: *Echinococcus granulosus*
qPCR: quantitative real-time polymerase chain reaction
CE: cystic echinococcosis
*E. coli*: *Escherichia coli*
cDNA: complementary DNA

## References

[1] Cardona GA, Carmena D (2013). A review of the global prevalence, molecular epidemiology and economics of cystic echinococcosis in production animals. Vet Parasitol.

[2] Zhang W, McManus DP (2006). Recent advances in the immunology and diagnosis of echinococcosis. FEMS Immunol Med Microbiol.

[3] Heath DD, Robinson C, Shakes T (2012). Vaccination of bovines against *Echinococcus granulosus* (cystic echinococcosis). Vaccine.

[4] Tomuş C, Zaharie F, Mocan L (2013). Minimal invasive treatment of abdominal multiorgan echinococcosis. Int Surg.

[5] Dalton JP, Mulcahy G (2001). Parasite vaccines - a reality. Vet Parasitol.

[6] Lightowlers MW, Flisser A, Gauci CG (2000). Vaccination against cysticercosis and hydatid disease. Parasitol Today.

[7] Barnes TS, Deplazes P, Gottstein B (2012). Challenges for diagnosis and control of cystic hydatid disease. Acta Trop.

[8] Fraize M, Sarciron ME, Saboulard D (2004). An in vitro model to evaluate the cytokine response in Echinococcus infections. Parasitol Res.

[9] Fraize M, Sarciron ME, Azzouz S (2005). Immunogenicity of two *Echinococcus granulosus* antigens EgA31 and EgTrp in mice. Parasitol Res.

[10] Vuitton DA (2003). The ambiguous role of immunity in echinococcosis: protection of the host or of the parasite?. Acta Trop.

[11] Urban JF, Madden KB, Svetić A (1992). The importance of Th2 cytokines in protective immunity to nematodes. Immunol Rev.

[12] Ding JB, Lin RY, Wen H (2003). Cloning and eukaryotic expression plasmid construct of *Echinococcus granulosus* 95 (Eg95) antigen gene. The Chinese people Zoonosis magazine.

[13] Liu XF, Ding JB, Li YJ (2012). The *Echinococcus multilocularis* 95 antigen T-B epitope analysis. Chinese Journal of Parasitic Disease.

[14] Alvite G, Esteves A (2009). *Echinococcus granulosus* tropomyosin isoforms: from gene structure to expression analysis. Gene.

[15] Saboulard D, Lahmar S, Petavy AF, Bosquet G (2003). The *Echinococcus granulosus* antigen EgA31: localization during development and immunogenic properties. Parasite Immunol.

[16] Fu Y, Martinez C, Chalar C (1999). A new potent antigen from *Echinococcus granulosus* associated with muscles and tegument. Mol Biochem Parasitol.

[17] Capron A, Capron M, Riveau G (2002). Vaccine development against schistosomia sis from concepts to clinical trials. Br Med Bull.

[18] Kalinna BH, McManus DP (1997). A vaccine against the Asian schistosome, *Schistosoma japonicum*: an update on paramyosin as a target of protective immunity. Int J Parasitol.

[19] Jounai N, Kobiyama K, Takeshita F, Ishii KJ (2012). Recognition of damage-associated molecular patterns related to nucleic acids during inflammation and vaccination. Front Cell Infect Microbiol.

[20] McManus DP, Loukas A (2008). Current status of vaccines for schistosomiasis. Clin Microbiol Rev.

[21] Nara T, Tanabe K, Mahakunkijcharoen Y (1997). The B cell epitope of paramyosin recognized by a protective monoclonal IgE antibody to *Schistosoma japonicum*. Vaccine.

[22] Esmaelizad M, Ahmadian G, Aghaiypour K (2013). Induction of prominent Th1 response in C57Bl/6 mice immunized with an *E. coli*-expressed multi T-cell epitope EgA31 antigen against *Echinococcus granulosus*. Folia Parasitol (Praha).

[23] Heath DD, Robinson C, Lightowlers MW (2012). Maternal antibody parameters of cattle and calves receiving EG95 vaccine to protect against *Echinococcus granulosus*. Vaccine.

